# Efficacy of Submucosal Sodium Tetradecyl Sulfate in the Soft Palate as a Treatment of the Mild Obstructive Sleep Apnea Syndrome: A Pilot Study

**DOI:** 10.1155/2012/597684

**Published:** 2012-01-24

**Authors:** Alberto Labra, Reyes Haro-Valencia, Angel-Daniel Huerta-Delgado, Ulises Jimenez-Correa, Francisco Sanchez-Narvaez

**Affiliations:** Clínica de Trastornos del Sueño, Facultad de Medicina, Universidad Nacional Autonoma de Mexico (UNAM), 06726 Mexico City, DF, Mexico

## Abstract

*Background*. As described by Mair et al. in 2001, snoreplasty, the application of sclerosant agents in the palate is a promising and cheap alternative to treat snoring. We decided to try this kind of therapy for the management of mild sleep apnea. *Study Design*. Experimental, longitudinal, prospective, nonrandomized, self-controlled pilot study. *Methods*. 11 patients were included, all of them with a polysomnographic study showing an Apnea-Hypopnea Index (AHI) from 5 to 20, and with a Müller maneuver showing only retropalatal collapse. *Results*. We found significant decrease in the number of apneas hypopneas and oxygen desaturation as well as in the snoring index (*P* < 0.05), although no differences were found in the number of arousals. *Conclusion*. Sclerosant agents might become a relevant part in the treatment of sleep apnea, in very well-selected patients.

## 1. Introduction

The obstructive sleep apnea syndrome (OSAS) is one of the most commonly found sleep disorders around the world. A number of studies show that it may be present in 5–20% of the adult population, and about 40 million Americans seem to be affected [1]. The obstruction in the upper airway may be present at one or many anatomical locations: the nasal septum, turbinates, tonsils, adenoids, soft palate, base of tongue, and even epiglottis, [2] and its presentation includes not only adults, given that children can be affected as well [3].

 Many authors have demostrated a strong association between OSAS and a high incidence of traffic accidents, becoming one of the most important risk factors, just following alcohol [4]. OSAS has also shown to be an important factor in the development of cardiovascular conditions such as systemic hypertension and stroke [5, 6].

 According to the International Classification of Sleep Disorders (ICSD, 2005), diagnosis of OSAS has to be suspected on patients complaining of snoring, astenia, cognitive disorders, and excessive daytime somnolence. Polysomnography should be performed on these patients in order to confirm the diagnosis, getting the apnea-hypopnea index (AHI) and rule out the presence of central or mixed apnea periods [5, 6].

 The site or sites of obstruction at the airway should be suspected during the physical examination. The use of imaging studies like cephalometric measures, computed tomography, and magnetic resonance have been proposed, but none of them have shown to be more effective. Flexible fiberoptic nasopharyngoscopy with Müller maneuver may be a more accurate method to determine the obstructive area, despite its usefulness is still controversial [7].

 There are many options for the OSAS treatment, according to its severity and to the site of obstruction. Medical treatment includes exercise programs, weight control, management of associated medical conditions (such as hypothyroidism and gastroesophageal reflux disease) mandibular advance devices, and nasal continuous positive airway pressure, which remains as the gold standard [8].

 Surgical treatments also include different approaches, and all of them remain controversial. Uvulopalatopharyngoplasty (UPPP) is the most commonly used technique. However, it is a surgical procedure performed under general anesthesia, which increases costs and risks. Laser-assisted uvulopalatoplasty (LAUP) can be performed at the office, under local anesthesia. Nevertheless, it has 2 important disadvantages: it is an expensive and very painful procedure [9]. The use of controlled temperature radiofrequency energy (Somnoplasty, Gyrus ENT, Bartlett, TN) is a relatively new kind of therapy. It could be used at the palate, turbinates, and base of tongue and it is performed at the office with a minimal or null discomfort. Again, its price is its main disadvantage [10].

 Brietzke and Mair, in 2001, reported the use of a widely known sclerosant agent, sodium tetradecyl sulfate (STS), as a painless and cheaper procedure (injection snoreplasty) to successfully treat snoring in 27 patients [11]. Only primary snoring was considered for the treatment, excluding OSAS patients. In the present study, we decided to try this injection snoreplasty to treat mild OSAS, but only when the obstruction site was located exclusively at the soft palate.

## 2. Methods

Eleven consecutive patients with the diagnosis of mild OSAS from the Sleep Disorders Clinic of the National University of Mexico were enrolled in this experimental, longitudinal, prospective, nonrandomized, and self-controlled pilot study, performed with the approval of the Ethics and Research Board of the General Hospital of Mexico. All patients underwent clinical assessment and full overnight sleep study (Alice 3, USA).

Our inclusion criteria included both genders, from 18 to 65 years old, with no metabolic or coagulation diseases, with small palatal tonsils (included between the anterior and posterior pillars), AHI from 5 to 20, and obstruction located at the palate. AHI was defined as the number of both, apneas (obstructive, mixed or central) and hypopneas, during the full night and divided by the number of hours the patient kept asleep, and snoring index (SI) was defined as the number of snoring events per hour of sleep. Only patients with body mass index (BMI) from 24 to 26 were included, trying to avoid obesity as a confusing factor. Pregnant women were excluded from the study, because the security range of STS has not been established in such cases. Septal abnormalities, turbinal hypertrophy, uvula longer than 1 cm, and base of tongue obstruction were also exclusion criteria, given that the aim of the study was to determine the usefulness of STS only at the palate.

Elimination criteria included patients who did not accept to participate in the trial and those who decided to stop their participation at any time.

 Confirmation of the obstruction site was achieved using the Müller maneuver, accepting only the cases with palatal collapse and eliminating those whose obstruction sites were located at the nose, base of tongue, lateral walls, or epiglottis. In a previous study, we found a concordance *kappa* test value of 0.9.

**Figure 1:**
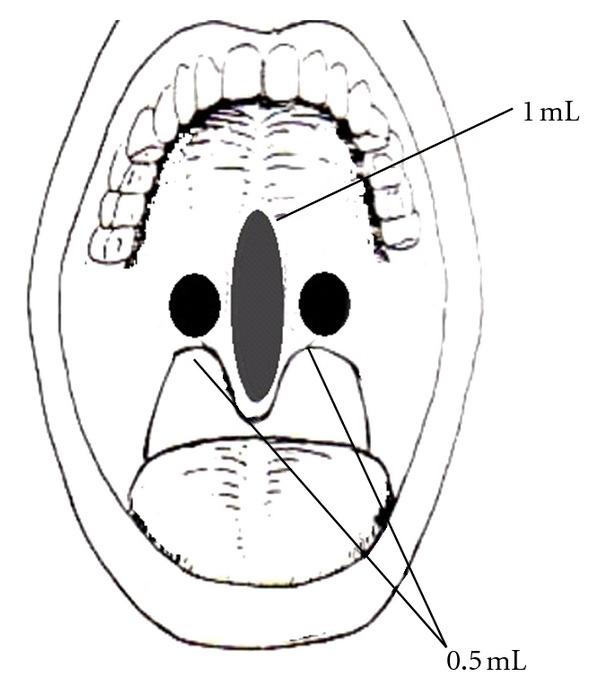
Injection procedure technique at the soft palate.

 Once the patients were properly selected, we injected 2 mL of 3% STS (Fibro-Vein 3%, STD Pharmaceutical Products LTD, Harreford, England). The whole procedure was performed at the office. Topical anesthesia was applied using 10% spray lidocaine, about 5 minutes before the procedure was performed. The injection technique was similar to the one described by Brietzke and Mair in 2001, but only 1 mL was injected submucosally at the midline and 0.5 mL on each side, using a tongue depressor to improve visualization of the area (Figure 1). Another difference from the original technique by Brietzke and Mair was that they used 1% STS instead of 3%, given that they were looking only for an improvement on snoring. We decided to use 3% STS in order to increase the possibility of volumetric tissue reduction, leading to an improvement on OSAS as well. All of the patients complained of a mild burning pain at the application site during a couple of minutes, but it was self-limited. All of them were sent home after 15 minutes, with a prescription for ketorolac tromethamine 10 mg, just in case that pain would be present. No antibiotics were prescribed. An appointment for followup was scheduled 6 months after the procedure, for clinical and fiberoptic assessment, and to perform a control overnight sleep study 6 months after the procedure.

AHI, snoring index, mean oxygen saturation, and arousals index were analyzed using a Student's *t*-test for related samples. Central tendency measures were performed as well, using SPSS 11.0 for Windows (LEAD Technologies, Chicago IL). Statistical significance was established at the *P* < 0.05 level.

## 3. Results

Descriptive statistics are shown in Table 1. The age of the 11 patients ranged from 22 to 62 years old, with a mean of 43.36 years old. 9 of them were males and 2 females. Only one patient complained of a small ulcer in the site of application, which required no treatment at all.

For the preoperative AHI, we found a range from 7.9 to 20 (mean 14.47), while the postoperative AHI ranged from 0 to 8 (mean 4.13). We found only obstructive apneas and hypopneas, and no central or mixed were found. Standard deviation was 5.156, the 95% Confidence Interval (CI) for the difference was 6.87/13.8, with a Student's *t* = 6.649, and a *P* < 0.001. All measures had 10 degrees of freedom and a 2-tailed value for the *t*-test.

Regarding preoperative snoring index, it ranged from 33 to 672 (Mean 149), and the postoperative range was from 4 to 114 (Mean 20.4), the 95% CI showed 24.21/233.51. Standard deviation was 155.776, for a Student's *t* = 2.744, and a *P* = 0.021.

On the mean oxygen saturation, we found preoperative values ranging from 81 to 88 (Mean 83.53), while the postoperative values ranged from 89 to 98 (Mean 91.63), with a 95% CI of −10.21/−5.97, standard deviation of 3.158, for a Student's *t* = 8.504, and a *P* < 0.001.

Finally, in terms of the number of arousals, we found that the preoperative mean was 148, and the postoperative mean was 86.6; there was a 95% CI of −17.38/140.11 and standard deviation of 117.218, for a Student's *t* = 1.736, and a *P* = 0.113, which is non statistically significant.

As a subjective measure we used the Epworth Sleepiness Scale, in order to evaluate day-time excessive sleepiness. We found a preoperative mean of 9.6364 (4–13), and postoperative mean of 8.2727 (5–12), for a *t* = 2.88 and a *P* = 0.016.

All the pre and posttreatment “Student's *t*-test” and “*P*” values are shown in Table 2.

We also asked the bed partner about the subjective improvement regarding snoring (improved, remained the same, or got worse). 10 patients improved, while only one referred that snoring had no changes. No patients got worse in this study.

In all cases, the last clinical and polysomnographic assessment was made 6 months after the initial procedure. After a followup of 6 months, we did not find any permanent complication, and patients as well as their bed partners were satisfied with the clinical results.

## 4. Discussion

Both objective and subjective outcome measures were used to assess the efficacy in this study. Hawthorne effect, that is, clinical improvement due only to the fact of being treated, represents a high risk on every self-controlled trials [12], so the elimination of day-time somnolence, headache, and other complaints should never be the best way to evaluate the success of any treatment modality. Nevertheless, we present numeric values that cannot be affected by the subjective perception of the patient: the AHI, the snoring index, the number of arousals, and the mean oxygen saturation level.

 The small number of patients is a weak issue in our study. The reason why we present a pilot study is that when we tried to estimate the sample size properly, we were not able to find in the English language literature, adequate statistical data that we could use, not even standard deviation. One of the main objectives of any pilot study is to provide descriptive statistics that may be used to calculate the sample size in future well-designed studies [12].

 All of the patients showed a significant improvement in the subjective variables, specially regarding snoring referred by the bed partner. All of them referred that the morning headache diminished importantly. Epworth Sleepiness Scale did not show significant changes, perhaps due to the fact that all of the patients had only mild sleep apnea, so day-time sleepiness was not a real complain on these patients.

 We were very careful to include in our study only patients whose obstruction site was located exclusively at the soft palate. This fact gives to the study strong internal validity, so we shall remind that similar results will only be reached when the selection criteria are fullfilled, including a very methodical search for the site of obstruction.

The statistical analysis shows a highly significant change when comparing the AHI, the mean levels of oxygen, and the snoring index. This statistical significance was very evident on both, the *P* value and the 95% confidence intervals of the difference. Despite these changes, no significant difference was found on the arousal index. However, we should keep in mind that the lack of statistical significance does not always mean a lack of biological one.

There is an antecedent regarding the effectiveness of sclerosant agents in the treatment of primary snoring, reported by Brietzke and Mair [11], but this cheap and painless therapy choice had never been tried to treat OSAS. Our results support the possibility of keeping it as an important tool when treating sleep breathing disorders but only when the site of obstruction is exclusively located at the soft palate, with mild OSAS, and in no obese patients, and as a primary treatment modality. In our series, after a followup of 6 months, a single application seems to be effective.

In 2008, Al-Jassim and Lesser [13], used injection snoreplasty in 60 primary snorers, as a part of their assessment, in order to rule out if the snoring was originated at the palate or anywhere else, so they could eventually decide if further and more aggressive palatal surgery could be indicated. They did not evaluate OSAS patients. We believe that the main reason why the surgical management of OSAS remains so controversial is the fact that the otolaryngologists keep looking for a surgery that cures every case of OSAS, even performing surgeries to patients who were not candidates for surgical management from the begining. We think that the key point in the managements of this kind of patients is to perform a complete preoperative evaluation, using every tool that we have available, including polysomnography, endoscopy, and a thorough clinical assessment.

## 5. Conclusions

The application of sodium tetradecyl sulfate in the soft palate (injection snoreplasty) has been proved as a treatment for primary snoring, and it may play an important role in the management of mild obstructive sleep apnea syndrome, but only when its origin lies exclusively at the soft palate. It represents a cheap and painless procedure, easily and quickly performed at the office, with no convalescence, and no complications in our study.

 We should keep in mind that the most important point when treating OSAS patients is an accurate preoperative evaluation and the concept of looking for the best surgical procedure should be changed for the concept of accurately finding the level or levels of obstruction, and treating the different obstructive areas as a whole, as a patient and not as an obstructed anatomic area.

 New studies, adequately designed and with a proper sample size, and with a longer followup must be performed in the next future, in order to determine the role of the injection snoreplasty in the treatment of moderate or severe OSAS, or when the site of obstruction is located at the base of tongue, either as a single procedure or as a part of a multilevel approach (which may include mandibular advance devices and other medical and conservative treatment modalities). Nevertheless, this procedure must be considered as a realistic alternative when treating mild OSAS if the site of obstruction is located exclusively at the soft palate.

## Figures and Tables

**Table 1:** Descriptive statistics.

	Number	Minimum	Maximum	Mean	STD
Age	11	22	62	43.3636	11.39537
AHI pre	11	7.9	20	14.4727	4.49513
AHI post	11	0	8	4.1364	2.32348
Snore index pre	11	33	672	149	185.45044
Snore index post	11	4	114	20.4	31.65691
O_2_ mean pre	11	81	88	83.5364	2.2686
O_2_ mean post	11	89	98	91.6336	3.31051
Arousals index pre	11	10	491	148	131.79378
Arousals index post	11	8	224	86.6364	58.41279
BMI	11	24	26	25.1727	0.69151

AHI: Apnea-Hypopnea Index. Pre: Preoperative. Post: Postoperative. O_2_: Oxygen. BMI: Body Mass Index.

**Table 2:** Student's *t*-test and *P* values.

	Student's *t*	*P* values
AHI	6.649	<0.001*
Snoring index	2.744	0.021*
Mean oxygen saturation	8.504	<0.001*
Arousals	1.736	0.113
Epworth sleepiness scale	2.88	0.016*

AHI: Apnea-Hypopnea Index. *indicates significant *P* values.

## References

[1] Piccirillo JF, Thawley SE, Cummings CW (1998). Sleep-disordered breathing. *Otolaryngology Head-Neck Surgery*.

[2] Poole MD, Bailey BJ (1993). Obstructive sleep apnea. *Head and Neck Surgery Otolaryngology*.

[3] Salib RJ, Sadek SA, Dutt SN, Pearman K (2000). Antrochoanal polyp presenting with obstructive sleep apnoea and cachexia. *International Journal of Pediatric Otorhinolaryngology*.

[4] American Thoracic Society (1994 ). Sleep apnea, sleepiness and driving risk. *American Journal of Respiratory and Critical Care Medicine *.

[5] Palomaki H, Partinen M, Juvela S, Kaste M (1989). Snoring as a risk factor for sleep-related brain infarction. *Stroke*.

[6] Snyder F, Hobson JA, Morrison DF, Goldfrank F (1964). Changes in respiration, heart rate, and systolic blood pressure in human sleep. *Journal of Applied Physiology*.

[7] Skatvedt O (1993). Localization of site of obstruction in snorers and patients with obstructive sleep apnea syndrome: a comparison of fiberoptic nasopharyngoscopy and pressure measurements. *Acta Oto-Laryngologica*.

[8] Anand VK, Ferguson PW, Schoen LS (1991). Obstructive sleep apnea: a comparison of continuous positive airway pressure and surgical treatment. *Otolaryngology*.

[9] Neruntarat C (2001). Laser-assisted uvulopalatoplasty: short-term and long-term results. *Otolaryngology*.

[10] Powell NB, Riley RW, Troell RJ, Li K, Blumen MB, Guilleminault C (1998). Radiofrequency volumetric tissue reduction of the palate in subjects with sleep-disordered breathing. *Chest*.

[11] Brietzke SE, Mair EA (2001). Injection snoreplasty: how to treat snoring without all the pain and expense. *Otolaryngology*.

[12] Dawson B, Trapp RG (2001). *Basic and Clinical Biostatistics*.

[13] Al-Jassim AH, Lesser THJ (2008). Single dose injection snoreplasty: investigation or treatment?. *Journal of Laryngology and Otology*.

